# First-principles computational investigation of nitrogen-doped carbon nanotubes as anode materials for lithium-ion and potassium-ion batteries†

**DOI:** 10.1039/c9ra03235e

**Published:** 2019-06-03

**Authors:** Chengxi Zhao, Yunxiang Lu, Honglai Liu, Linjiang Chen

**Affiliations:** Key Laboratory for Advanced Materials and Department of Chemistry, School of Chemistry and Molecular Engineering, East China University of Science and Technology Shanghai China yxlu@ecust.edu.cn; Department of Chemistry and Materials Innovation Factory, University of Liverpool Liverpool UK linjiang.chen@liverpool.ac.uk; Leverhulme Research Centre for Functional Materials Design, Materials Innovation Factory and Department of Chemistry, University of Liverpool Liverpool UK

## Abstract

Significant research efforts, mostly experimental, have been devoted to finding high-performance anode materials for lithium-ion and potassium-ion batteries; both graphitic carbon-based and carbon nanotube-based materials have been generating huge interest. Here, first-principles calculations are performed to investigate the possible effects of doping defects and the varying tube diameter of carbon nanotubes (CNTs) on their potential for battery applications. Both adsorption and migration of Li and K are studied for a range of pristine and nitrogen-doped CNTs, which are further compared with 2D graphene-based counterparts. We use detailed electronic structure analyses to reveal that different doping defects are advantageous for carbon nanotube-based and graphene-based models, as well as that curved CNT walls help facilitate the penetration of potassium through the doping defect while showing a negative effect on that of lithium.

## Abstract 2

First-principles simulations reveal atomistic mechanisms for adsorption and migration of lithium and potassium on nitrogen-doped carbon nanotubes.

## Graphical abstract

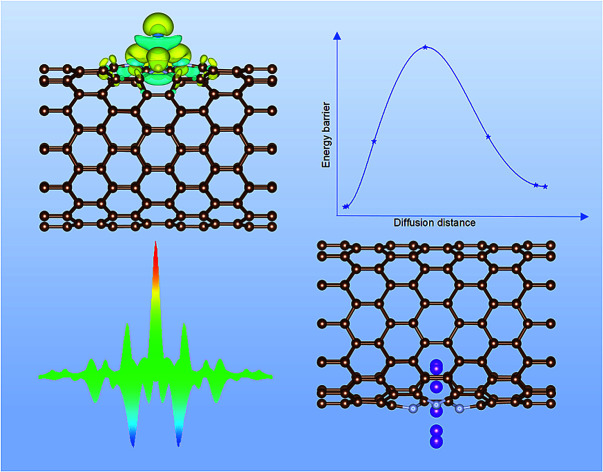

## 1. Introduction

With the rapid development of the electronics market, the demand for energy has rocketed up, giving rise to acute needs for more, improved energy storage methods. Lithium-ion batteries (LIBs) have been the predominant choice for electronic storage devices, owing to their high power and energy density, since they were firstly commercialized in 1990s by Sony Corporation.^1^ However, lithium is neither regarded as an abundant element nor are its resources evenly distributed around the world.^2,3^ This has motivated the pursuit of alternative ion batteries based on earth-abundant alkali metals, such as sodium (Na) and potassium (K).

Sodium-ion batteries (SIBs) and potassium-ion batteries (PIBs) have been considered promising alternatives to LIBs on the basis of material abundance and that Na and K are the closest alkali metals to lithium. However, the common graphite anodes used in LIBs cannot be used in SIBs directly, because the Na–C system lacks suitable binary intercalation compounds.^4^ Although SIBs have, over the years, achieved significant performance improvements in capacity, cycle life, and rate capability,^5^ it is PIBs that have gained intensified spotlight in the recent years for their own advantages,^6–8^ such as less complicated interfacial reactions, higher ionic conductivity,^2^ low price,^9^ environmental friendliness during application and recycling.^10,11^ Although the potassium ion has a larger ionic size compared with the sodium ion, it has been shown that potassium ions can be inserted into different types of carbon based materials more easily.^1,12^ Furthermore, PIBs can offer a higher working voltage, because of the lower redox potential of K than Na.^13^

To date, a variety of carbon nanomaterials, *e.g.*, graphene,^14^ carbon nanofibers,^15^ and carbon nanotubes (CNTs),^16^ have been studied as electrode materials. They have potential application as future electronic devices.^17^ Among them, nitrogen-doped (N-doped) carbon materials are attractive, because the electronegativity of nitrogen is larger than that of carbon (3.5 *vs.* 3.0; arbitrary units).^18^ Significant enhancements in battery performance may be further achieved because of the following two aspects: first, defects formed as a result of doping can benefit the diffusion of Li/K ions; second, the incorporation of nitrogen atoms into the carbon material yields stronger interactions between the alkali metal ions and the material.

In 2011, Cui *et al.*^18^ showed that N-doped graphene nanosheets were a promising candidate for anode materials in high-rate LIBs, as a result of their high reversible capacity, excellent rate performance, and significantly enhanced cycling stability. Subsequently, heavily N-doped porous carbons, prepared from a metal–organic framework, were reported as anode materials, showing a high capacity of 2132 mA h g^−1^.^19^ Very recently, Pint and co-workers^20^ demonstrated that N-doping of few-layered graphene can improve the storage capacity of PIBs from a theoretical maximum of 278 mA h g^−1^ to over 350 mA h g^−1^, comparable with the typical anode capacity in commercial LIBs.

CNTs, first discovered by Iijima in 1991,^21^ possess a computed stoichiometry specific capacity as high as 372 mA h g^−1^ (MC_6_)^22^ and can adsorb alkali metals such as Li, K, Cs, and Rb.^1^ However, these atoms hardly diffuse into the inside of CNTs,^23^ limiting the capacities of such batteries. To improve the anode performance of CNT for ion batteries, several strategies—including ball-milling,^24^ doping,^25^ and point defects^26^—have been applied to achieve desirable properties and push the capacity limit. In a previous study, Choi *et al.* reported that N-doped CNTs (NCNTs) contained wall defects through which Li ions could diffuse into the interwall spaces as storage regions, giving rise to a capacity as high as 3500 mA h g^−1^.^27^ First-principles calculations indicated that N-doping had significant effects on the diffusion of Li atoms in CNTs.^28^ Despite these attempts, knowledge about PIBs and LIBs using NCNTs as anodes is still limited.

In this work, we demonstrate that N-doped CNTs could be used as a potential anode for PIBs from first-principles. Three different NCNTs—namely, graphitic (NQ), pyridinic (N6), and pyrrolic (N5) CNT structures—with various tube diameters were investigated. The binding energies of Li/K atoms adsorbed on the NCNTs and the energy barriers to their diffusion into the NCNTs were examined. Analyses of electron density difference (EDD), noncovalent interaction index (NCI), and (partial) density of states (DOS) were performed to provide insights into the mechanisms at play.

## 2. Theoretical methods

Periodic density functional theory (DFT) calculations were carried out with the Vienna Ab initio Simulation Package (VASP) version 5.3.5.^29–32^ The projector augmented-wave (PAW) method was applied to describe the electron–ion interactions.^33^ Generalized gradient approximation (GGA) with the Perdew–Burke–Ernzerhof (PBE) exchange-correlation functional was adopted to treat electron interaction energy.^34,35^ Grimme's semi-empirical DFT-D3 (ref. 36) scheme was used here to give a better description of long range interactions; the latest Becke–Johnson damping functions^37,38^ for the DFT-D3 method were adopted, which were shown to give reliable results in potassium-ion-containing systems.^39^ All calculations were treated as spin-unrestricted.

A kinetic-energy cutoff of 450 eV was used to define the plane-wave basis set, after initial basis set dependence testing, in agreement with previous studies of similar systems in the literature.^40^ During geometry optimizations, the Hellmann–Feynman force convergence criterion on each atom was set to smaller than 0.02 eV Å^−1^ and convergence threshold of self-consistency was set to 10^−5^ eV in total energy. The electronic Brillouin zone was sampled by Gamma-centered Monkhorst–Pack^41^ grids using 1 × 1 × 5 *k*-points. The electronic density of states (DOS) was calculated on a denser grid with 1 × 1 × 11 *k*-points. The climbing-image nudged elastic band (CI-NEB) method^42,43^ was employed to identify and characterize minimum-energy pathways for Li/K migrations and quantify the associated energy barriers.

**Fig. 1:**
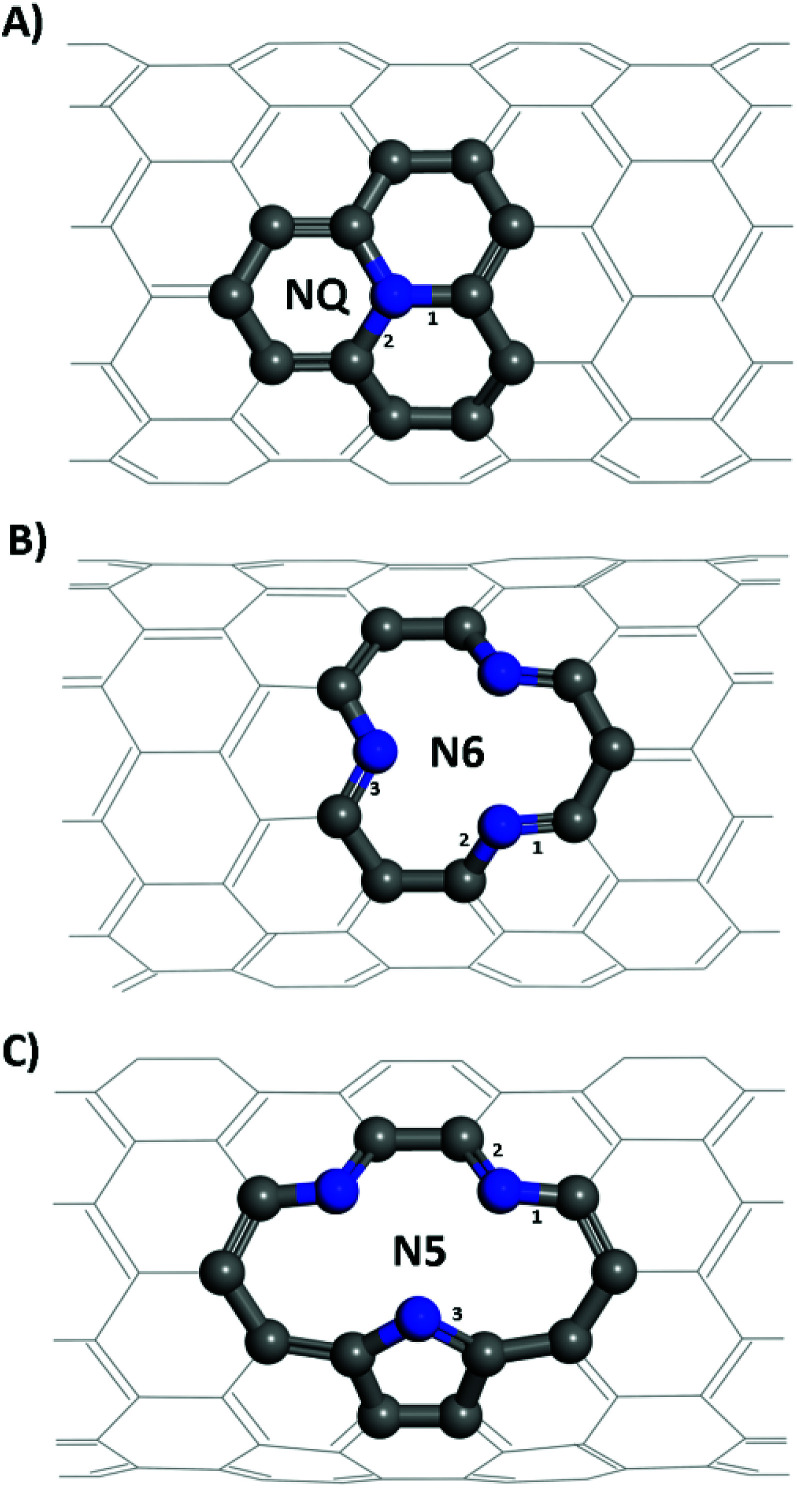
Structures of (A) graphitic like N-doping, NQ CNT, (B) pyridinic like N-doping, N6 CNT, (C) pyrrolic like N-doping, N5 CNT. Grey and blue atoms represent carbon and nitrogen of the doping site, respectively; the rest of the CNT is in grey with the backside of it omitted for clarity.

Structural models of the NQ, N5 and N6 CNTs were all of the zig-zag type (Fig. 1), which is a stable form and have been widely used in the literature.^28,44^ It is worth noting that the N5 CNT structure and the N6 CNT structure have the same molecular formula thus the same doping ratio. For all systems, the CNT was periodically repeated along its axial (*z*) direction and was separated from its periodic images in the other two directions (x, y) perpendicular to its axial direction by a vacuum layer of at least 15 Å in thickness. All the simulation boxes were of the length around 12.8 Å in the z direction, which was found to be sufficiently large according to our tests.

## 3. Results and discussion

### 3.1 Structure of CNTs and NCNTs

A CNT, which could be considered as formed by rolling up a section of a graphene sheet, can be characterized by the roll-up vector **C**_**h**_:

1 **C**_**h**_ = *n***a**_**1**_ + *m***a**_**2**_

where *n* and *m* are integers, and **a**_**1**_ and **a**_**2**_ are graphene's lattice vectors; the CNT is then denoted by (*n*,*m*). The structures of zig-zag, single-walled CNTs and NCNTs with five different diameters—*i.e.*, (8,0), (9,0), (10,0), (11,0), (12,0)—were studied. All supercells in the tube axial direction were around 12.8 Å after optimization, consistent with previous modelling results.^28,45^ The C–C bond lengths in the thinnest CNT deviates the most from those of graphene as the smaller the tube diameter the larger the curvature and the greater the surface strains.

From Table 1, it is seen that bond lengths in pristine, N5 and N6 CNTs do not differ markedly with the varying tube diameter, whereas slightly more pronounced (approx. 1%) differences are observed for NQ CNT. The C–N bonds (type 3 in Fig. 1C; 1.415–1.420 Å) in the pyrrole ring of N5 CNT are considerably longer than those (types 1–3 in Fig. 1B, and types 1 and 2 in Fig. 1C; 1.334–1.353 Å) in the pyridine rings of N5 and N6 CNTs, which indicates that the pyrrolic N atoms to some extent deviate from the regular pentagon point.

**Table 1:** Bond lengths for studied systems^a^

System	Bond^b^	CNTs type				
		(8,0)	(9,0)	(10,0)	(11,0)	(12,0)
Pristine	1(C–C)	1.418	1.420	1.422	1.421	1.422
	2(C–C)	1.435	1.432	1.430	1.429	1.428
NQ	1(C–N)	1.388	1.396	1.394	1.403	1.403
	2(C–N)	1.426	1.419	1.420	1.420	1.413
N6	1(C–N)	1.339	1.340	1.343	1.343	1.343
	2(C–N)	1.353	1.352	1.351	1.350	1.350
	3(C–N)	1.336	1.335	1.335	1.335	1.334
N5	1(C–N)	1.345	1.346	1.346	1.346	1.347
	2(C–N)	1.342	1.343	1.342	1.341	1.341
	3(C–N)	1.415	1.418	1.420	1.420	1.420

a Bond lengths are given in angstrom.

b Bonds are numbered as shown in Fig. 1.

### 3.2 Adsorption of lithium and potassium atoms on CNTs

To investigate the effects of N-doping on the key performance metrics for CNTs acting as a battery electrode, we first studied the binding energetics and mechanisms of Li and K adsorbed on the above described structural models of pristine and N-doped CNTs.

#### Pristine CNT

The adsorption of Li/K atoms on both the outside and the inside of the CNT was examined, and the binding energies of the alkali metal (AM) atoms with the CNTs were evaluated *via* the following equation:

2 *E*_b_ = *E*_CN*x*_ + *E*_AM_ − *E*_CN*x*−AM_

where *E*_CN*x*_, *E*_AM_, *E*_CN*x*−AM_ are the energies of the CNT, isolated AM atom, and CNT with adsorbed AM atom, respectively. It should be noted that both an isolated AM atom^46^ and the AM in its stable bulk phase^44^ have been commonly used as the reference state. Here, we used the isolated AM atoms to allow for a direct derivation of the binding energy.

**Fig. 2:**
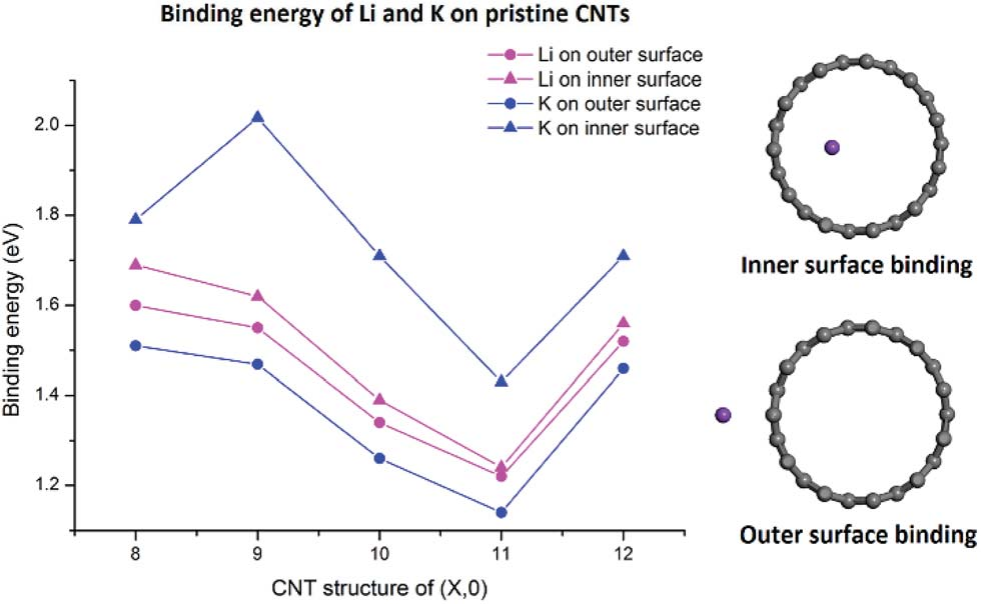
Binding energies of Li and K adsorbed on the inner and outer surfaces of the pristine CNT.

As shown in Fig. 2, both Li and K atoms exhibit a larger binding affinity with the inner surface than with the outer surface. We note that our calculations included dispersion corrections, using the DFT-D3(BJ) method, which is known to be important for adsorption energy calculations in similar systems.^39^ Our own test calculations and a previous report^45^ show that non-dispersion-corrected DFT can lead to a contradicting conclusion that Li/K binding is stronger on the outer surface.

The binding energies of the Li atom adsorbed on both sides of the CNT surface and those of the K atom on the outer surface decrease with the increasing tube diameter from (8,0) to (11,0), while CNT (12,0) yields a larger binding energy relative to (11,0) and (10,0), in agreement with previous studies.^45,47^ The adsorption of K atom on the inner CNT surface largely follows the same trend, except that the K–CNT binding energy peaks at CNT (9,0). We attribute this to the larger K atom experiencing increased repulsion from CNT (8,0) with the smallest tube diameter in the series, hence lowering the binding affinity. Notably, in all the CNTs with the varying tube size, the K atom binds more strongly with the inner surface compared to the Li atom, whereas the opposite is true with the outer surface.

#### Nitrogen-doped CNTs (NCNTs)

**Fig. 3:**
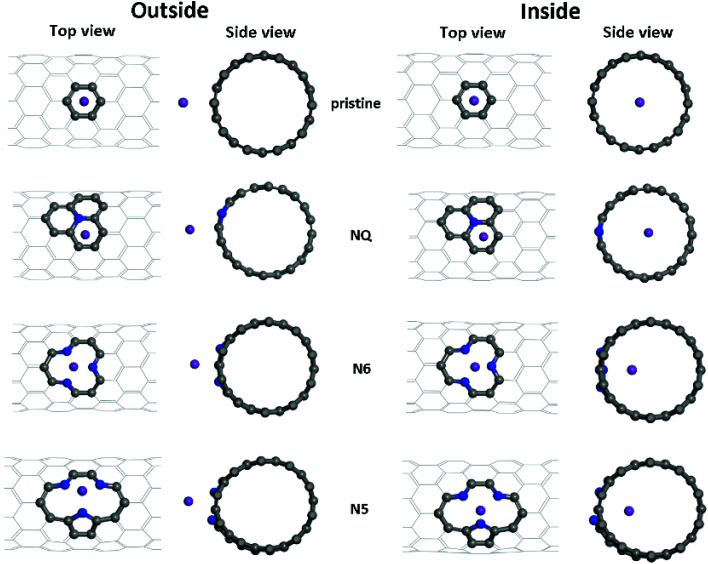
Different adsorption configurations of K/Li on pristine, NQ, N6 and N5 CNTs. Shown here are the optimized adsorption complexes for the K atom on (10, 0) CNTs.

In CNT-based ion batteries, both internal and external surfaces of the CNTs contribute to ion storage, with the latter playing a larger role.^1^ N-doping has been a widely adopted strategy to enhance the AM–CNT binding affinities, hence improving the battery capacity. Here we study Li/K binding with three types of N-doping sites—graphitic-, pyrrolic- and pyridinic-like doping—for NCNTs in three different tube sizes: (8,0), (10,0) and (12,0); see Fig. 3 and Table 2.

**Table 2:** Calculated binding energies for a single alkali-metal atom adsorbed on the pristine CNT and three types of N-doped CNTs, both on the outer and inner surfaces^a^

	Li		K	
	*E*_b,out_	*E*_b,in_	*E*_b,out_	*E*_b,in_
(8,0)				
Pristine	1.60	1.69	1.51	1.79
NQ	1.47	1.58	1.33	1.62
N6	4.88	3.59	3.44	2.57
N5	5.02	b	3.75	2.70
(10,0)				
Pristine	1.34	1.38	1.26	1.72
NQ	1.23	1.09	1.15	1.62
N6	4.96	3.77	3.47	2.76
N5	5.01	3.71	3.73	2.81
(12,0)				
Pristine	1.52	1.56	1.46	1.71
NQ	1.06	1.00	1.08	1.45
N6	4.80	3.70	3.29	2.61
N5	4.79	3.60	3.49	2.63

a Energies are given in eV.

b No stable adsorption configuration was found.

For zig-zag, single-walled CNTs, there exists only one possible conformation for the graphitic-like (NQ) doping and one for the pyridinic-like (N6) doping. However, the pyrrolic-like (N5) doping could adopt two different conformations (see Fig. S1†). On the basis of the formation energies calculated for the two conformations of N5 CNT (Fig. S1†), the thermodynamically more favorable conformation, as shown in Fig. 1 and 3, was used to represent N5 CNT throughout this study. The NCNT models used in this study are the same as, or similar to, those that have been studied theoretically or observed experimentally in the literatures.^28,44,48–50^

Optimized adsorption configurations of Li/K on both the inner and the outer surfaces of pristine/NQ/N6/N5 CNTs are shown in Fig. 3, with corresponding binding energies listed in Table 2. For both pristine and NQ CNTs, the Li/K atom is adsorbed on top of the aromatic ring, no matter with the outer or inner surface of the CNT. The NQ doping type is electron rich,^51^ leading to a weakened binding of Li and K, compared with the pristine CNT. Strong binding of AMs onto CNTs enhances the performance of electrodes because unfavorable formation of AM clusters is hindered, thus facilitating high AM adsorption amounts by the CNTs.^40^ Therefore, NQ CNTs are rarely a candidate for battery electrodes, in line with findings for 2D graphene systems.^40^ The decrease of binding of Li/K in NQ CNT (8,0) and (10,0) is relatively small because the AM atom is pushed away from the NQ doping site and drawn towards the other side of the CNT.

For Li/K adsorption on the N6 and N5 CNTs, the AM atom is energetically favorable to adsorb at the doping site and the binding energies are significantly increased, compared to the pristine CNT (Table 2). For example, the binding energies of Li and K adsorbed on outer surface of CNT (10,0) are 1.34 and 1.26 eV, respectively, which increase to 5.01 and 3.73 eV in N5 CNT (10,0). In addition, the effects of tube size on the AM binding with N6/N5 CNTs are more pronounced for Li than K and more on the outer surface than the inner surface.

Charge transfer characteristics were determined for Li/K adsorption on CNT (10,0) and NCNTs (10,0), using Bader charge analysis,^52^ and are listed in Table S1.† Our results show that the charge transfer between the AM and the pristine CNT and NQ CNT is more significant than that between the AM and the nitrogen-decorated vacancy of N5/N6 CNT, which is observed for both Li and K. The large charge transfer (0.82–0.96*e*) in these adsorption complexes indicates that the bonding between the AM atom and the CNTs has ionic characteristics and is dominated by electrostatic interactions.

**Fig. 4:**
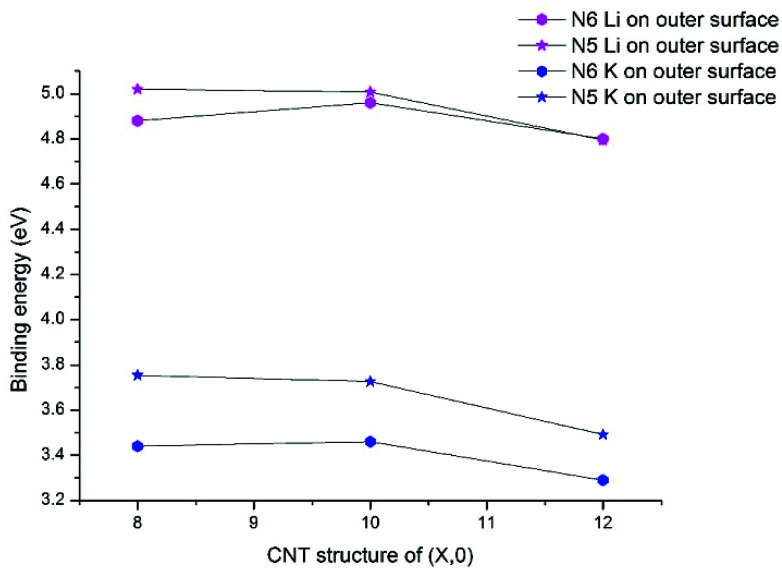
Binding energies of K and Li adsorption on the surfaces of N6 and N5 doped CNTs with the different tube diameters.

Previous research on N-doped graphene systems concluded that the pyridinic-like (N6) doping is the most effective type for alkali-ion.^53,54^ Our results show that such a conclusion does not directly translate to N-doped CNTs. In Fig. 4, we compare the binding energies for Li/K with N5/N6 CNT, all on the outer surfaces. Clearly, the pyrrolic-like (N5) doping outperforms the N6 doping for almost all cases, except that the Li binding with N5 CNT (12,0) is slightly less favorable than with N6 CNT (12,0) by 0.01 eV. The Li/K binding strengths with the (8,0) and (10,0) NCNTs are very similar for both N5 and N6 doping types, though the trends are different. That is, going from (8,0) to (10,0), the AM binding with N5 CNT decreases slightly while the AM binding with N6 CNT increases slightly. By contrast, moving on to (12,0), all binding strengths decrease markedly. Nonetheless, the binding energy of K–N5 CNT (12,0) is still 0.2 eV larger than that of K–N6 CNT (12,0). These results cast new insight into designing PIBs: while the N6 doping type has been recommended for the planar graphene,^40,44^ the N5 doping type can be more beneficial for curved surfaces.

### 3.3 Electronic structure analyses

**Fig. 5:**
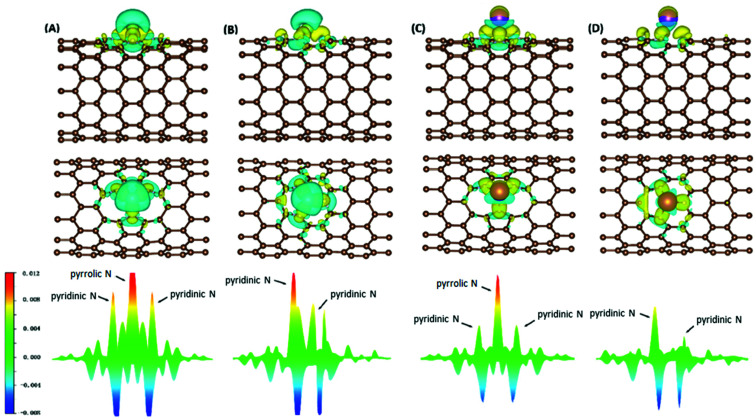
Differential electron densities (A) Li atom on N5 CNT, (B) Li atom on N6 CNT, (C) K atom on N5 CNT, (D) K atom on N6 CNT: top, side view; middle, top view; bottom, electron density differences in the plane determined by the three N atoms. For isosurfaces, cyan and yellow indicate electron depletion and accumulation, respectively; the isovalue is taken to be 0.0015 e Å^−3^.

Electron density difference (EDD) analyses were performed for selected systems, with differential electron densities shown in Fig. 5, in which the yellow iso-surfaces indicate regions with increased electron densities while the cyan ones indicate regions with decreased electron densities. The EDD plots were obtained by subtracting the electron densities of the isolated NCNT and isolated AM atom (*ρ*_NCNT_ and *ρ*_AM_, respectively) from the electron density of the AM@NCNT complex (*ρ*_AM@NCNT_):

3 Δ*ρ* = *ρ*_AM@NCNT*x*_ − *ρ*_NCNT*x*_ − *ρ*_AM_

For the Li–NCNT systems (Fig. 5A and B), increased electron densities are concentrated in regions between the Li atom and the N atoms, with decreased electron densities in the immediate vicinity, indicating marked charge transfer from Li to NCNTs. Similar features are also clear in the K–NCNT systems (Fig. 5C and D). Our EDD results show that the bonding between the AM atoms and the NCNTs has strong ionic characteristics, corroborating the Bader charge analysis described above, and that the electron redistributions between N5/N6 CNTs and Li are more significant than those between N5/N6 CNTs and K. In the AM–N5 CNT systems (Fig. 5A and C), the net gain of electronic charge on the pyrrolic N atom is more significant than those of the other two pyridinic N atoms. In the case of N6 CNT (Fig. 5B and D), the AM atom forms almost equally strong bonding with two pyridinic N atoms, hence the overlapping of the corresponding peaks in Fig. 5 (bottom), whereas the bonding with the third pyridinic N atom is considerably weaker.

To elucidate the nature of the inter-molecular interactions involved in the AM–NCNT systems, we use Non-Covalent Interactions index (NCI) analysis to probe and facilitate the visualization of both strong chemical bonds and weak interactions. The NCI index is the reduced density gradient *s* of the electron density *ρ*:4
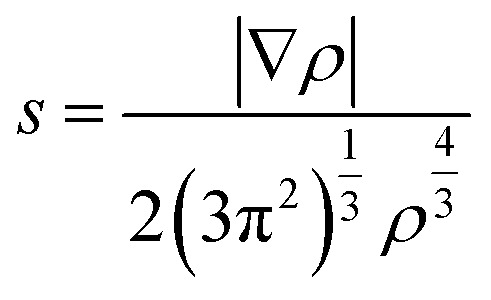


An isosurface of *s* determines the spatial area of the interaction, while the sign of the second eigenvalue (*λ*_2_) of the electron-density Hessian matrix distinguishes whether the interaction is attractive (*λ*_2_ < 0) or repulsive (*λ*_2_ > 0). In Fig. S2,† NCI identifies much stronger ionic characteristics of the interactions between Li and NCNTs, compared to those between K and NCNTs which are suggested to have a predominant nature of weak van der Waals interactions. It is also clear from Fig. S2† that the pyrrolic N atom binds more strongly with both Li and K than the pyridinic N atom does. These NCI results provide further support to the above discussions based on the differential electron density analyses.

### 3.4 Migration of Li and K atoms into the NCNTs

Migration of AM atoms into CNTs is an important performance metric for ion batteries, as the ability to utilize the inside space of CNTs will create additional storage space for enhanced capacity.^27^ Therefore, we investigated the diffusion of Li/K atom into selected N-doped CNTs, through the doping site, using first-principles DFT coupled with the climbing-image nudged elastic band (CI-NEB) method. Six NCNTs—N5/N6 CNT (8/10/12,0)—were studied for both Li and K migrating into the NCNTs, yielding twelve systems in total.

**Fig. 6:**
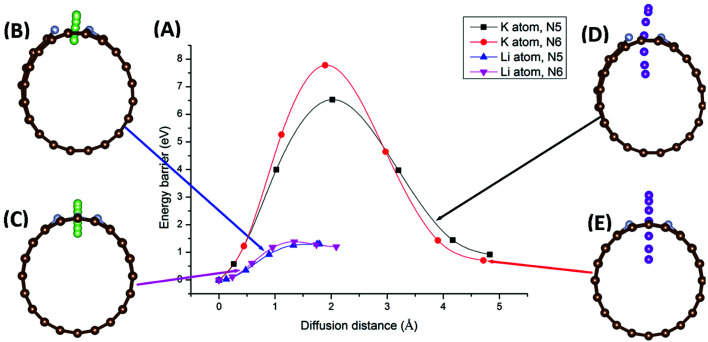
Minimum-energy pathways for Li and K migration into N5 CNT (10,0) and N6 CNT (10,0), through the doping site: potential energy profiles (A) of the minimum-energy pathways for Li penetrating N5 CNT (B), Li penetrating N6 CNT (C), K penetrating N5 CNT (D), and K penetrating N6 CNT (E).

Minimum-energy pathways, determined by the CI-NEB calculations, for Li/K atom penetrating N5 CNT (10,0) and N6 CNT (10,0) are shown in Fig. 6. The energy barrier to the diffusion of Li atom into N5 CNT is 1.30 eV, which is slightly lower than that for N6 CNT (1.38 eV). By contrast, the K atom experiences significantly larger diffusion barriers from both N5 CNT and N6 CNT. The energy barrier of K penetrating N5 CNT is as high as 6.53 eV and further increases to 7.78 eV in the case of N6 CNT. The migration distances for K are significantly longer than those for Li due to the adsorption sites of K being further away from the wall of the NCNT. The large diffusion barriers of K, combined with their required long penetration pathways, suggest that single-vacancy defects may not allow K atoms to access the internal space of NCNTs, hence unable to maximize the storage capacity of batteries based on them. One possible solution to this may be incorporation of multiple-vacancy defects to CNTs, rendering the apertures of doping sites large enough for easy K diffusion. In passing, we note that the diffusion paths of Li and K in N5 CNT (Fig. 6B and D) are not as straight-line as those in N6 CNT, because the pyrrolic N atom interacts strongly with the AM atom, which is pulled toward the pyrrolic N atom during the migration through the doping site.

**Fig. 7:**
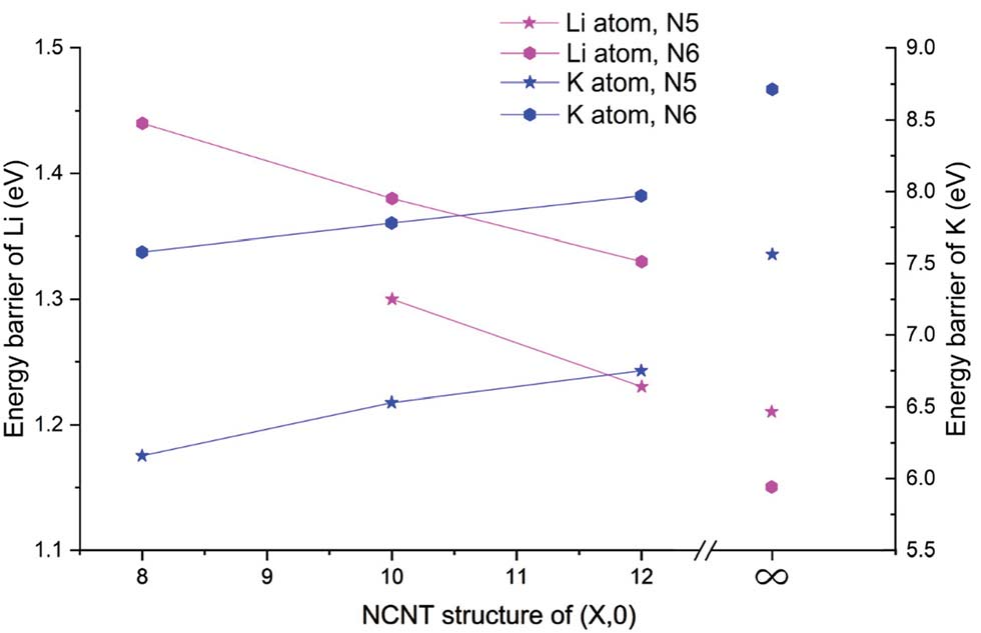
Energy barriers, determined from minimum-energy pathways, of the AM atom penetrating the NCNT with a different tube diameter. The vertical axis for the energy barriers of Li is on the left while the vertical axis for the energy barriers of K is on the right; different scales are used. Note that the energy barrier of Li penetrating N5 CNT (8,0) was not determined because no stable adsorption site was found for Li inside the NCNT.

Next, we determined diffusion barriers for Li/K penetrating the sidewall of NCNTs as a function of the tube diameter and results are reported in Fig. 7. As the NCNT tube diameter increases, the energy barrier to Li penetration decreases from 1.44 to 1.33 eV for N6 CNTs and from 1.30 to 1.23 eV for N5 CNTs. By contrast, the penetration energy barriers for K increases, with increasing tube diameter, from 7.58 to 7.97 eV for N6 CNTs and from 6.16 to 6.75 eV for N5 CNTs. Across the tube diameter range (8,0) to (12,0), the pyridinic N6 doping type consistently imposes a larger energy barrier to AM diffusion than the pyrrolic N5 counterpart, with the energy differences being approximately 0.1 and 1.3 eV for Li and K, respectively. Furthermore, we probed the AM diffusion profiles in the case of infinite tube diameter, using models based on a single graphene sheet incorporated with either an N5 or N6 doping defect (see Fig. S3†). Interestingly, the Li atom experiences a slightly larger energy barrier (1.21 *vs.* 1.15 eV) when penetrating the N5 defect than when penetrating the N6 defect, in a reversed ordering compared to the NCNTs. For K migration, the N5 doping type continues to be the favourable one for graphene, yielding a lower energy barrier than the N6 doping type (7.56 *vs.* 8.70 eV). However, these energy barriers are higher than those of the NCNTs. Our results suggest that N5 and N6 CNTs with a smaller tube diameter, therefore having a larger curvature, are better able to facilitate the migration of K through the doping defect.

### 3.5 Discussion

**Fig. 8:**
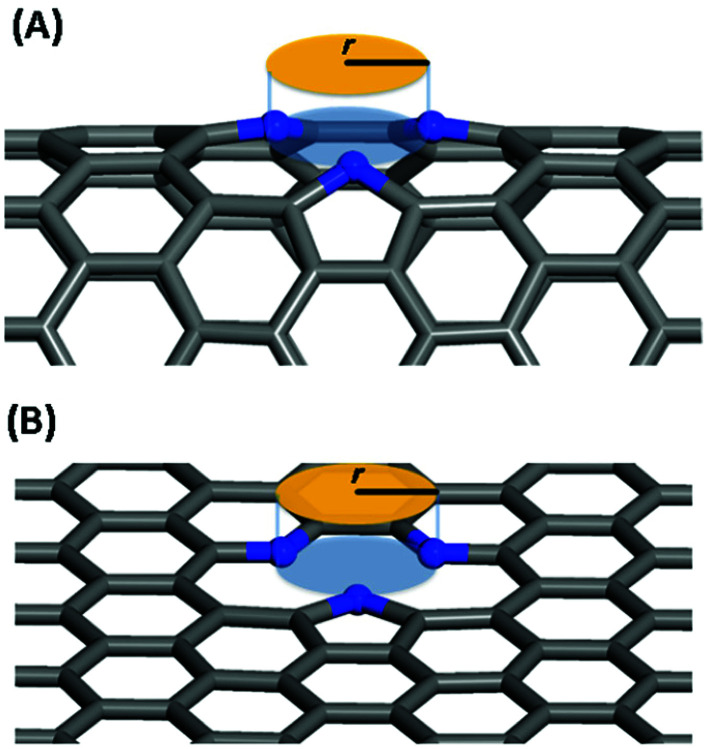
Schematic diagram for the definition of the aperture created by the doping defect: (A) N5 CNT, (B) N5 graphene.

In this work, we systematically studied the effects of the pyrrolic N5 and pyridinic N6 doping defects on the adsorption and diffusion of Li and K for NCNTs of different tubular sizes ranging from (8,0) to (12,0). Our results reveal that N5 CNTs are a more suitable doping strategy to allow for higher Li/K storage capacities, because of the stronger binding affinity and lower penetration energy barriers toward Li and K, compared to their N6 counterparts. We show that it is energetically advantageous to keep the tubular size of NCNT small for enhanced Li/K adsorption energies. Increased tube diameters lead to significantly increased energy barriers to K penetration, albeit slightly lowering the penetration energy barriers for Li. To elucidate how the tube size, and its resultant curvature of the tube wall, influences the size of the aperture created by the doping defect (Fig. 8), we calculated the diameter (*d*) of the circle and the area (*S*) of the triangle, both as defined by the three N atoms (Table 3):5
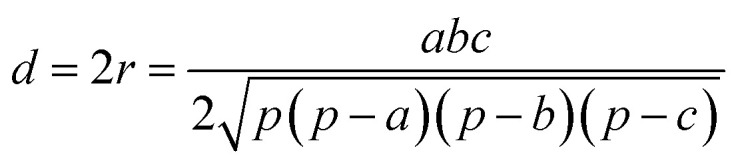
6
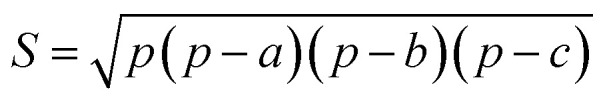
where *a*, *b* and *c* are the distances between each pair of the three N atoms, and *p* is equal to (*a* + *b* + *c*)/2; *r* is the radius of the circle.

**Table 3:** Geometrical characteristics of the apertures in different N5/N6 CNTs and N-doped graphene

	Doping type	N–N′ distance (Å)			Diameter (Å)	Area (Å^2^)
		*a*	*b*	*c*	*d*	*S*
NCNT (8,0)	N5	2.75	2.87	2.75	3.23	3.37
	N6	2.80	2.66	2.66	3.13	3.16
NCNT (10,0)	N5	2.88	2.69	2.69	3.19	3.27
	N6	2.77	2.65	2.65	3.11	3.13
NCNT (12,0)	N5	2.88	2.65	2.65	3.15	3.20
	N6	2.74	2.64	2.64	3.09	3.09
Graphene	N5	2.92	2.37	2.37	3.01	2.72
	N6	2.61	2.61	2.61	3.01	2.94

As the tube diameter increases, going from (8,0) to (12,0) and further to infinity (as represented by graphene models), the aperture created by the doping defect becomes smaller and smaller (Table 3). The diameter of the aperture circle decreases from 3.23 to 3.01 and from 3.13 to 3.01 in the case of N5 and N6 doping, respectively. The area of the aperture triangle decreases correspondingly. The apertures generated by the N5 type of defect are consistently larger than those generated by the N6 type of defect. These geometrical features explain well the above-discussed energetic and kinetic differences arising from the different tube sizes and from the different doping defects. We further note that out-of-plane protrusions are produced by the pyrrolic N5 doping defects; the larger the curvature of the NCNT wall, the greater the extent to which the pyrrolic ring protrudes out of plane. By comparison, the pyridinic N6 doping defects do not deviate markedly from the pristine CNT. We have also observed, from the CI-NEB calculations, that the pyrrolic rings of N5 defects are more mobile during the passing of the AM atom, suggesting a cooperative behavior that is desirable for guest diffusing through an aperture of comparative size.

## 4. Conclusions

In summary, we systematically investigated the effects of the tubular size and N-doping type of CNTs on the adsorption of Li and K at the doping site as well as their penetration through the defect aperture. Using first-principles calculations, we carried out detailed study and analysis of adsorption energetics, electronic structures of the adsorption complexes, and minimum-energy pathways for AM migration through the defects. We show that the pyrrolic-type defects outperform the pyridinic counterparts in both offering stronger binding affinities toward Li and K and allowing for easier passage of Li and K through the defect. Interestingly, this contradicts the frequently-reached conclusion for graphene-based systems that pyridinic defects are preferred over pyrrolic ones. Our study reveals that the curved NCNT walls make pyrrolic defects advantageous, because of their protruded configurations with enhanced binding strengths as well as enlarged and, to some extent, cooperative defect apertures. These beneficial effects combined facilitate strong adsorption interactions, high capacities and good mobilities of alkali metals in NCNTs. We hope that the molecular-level insights obtained, as well as the computational protocol demonstrated, in this study will help improve our understanding in the important performance metrics of materials for batteries and motivate further development in computational methods that will enable *in silico* prediction, and even *a priori* design, of new battery materials.

## Conflicts of interest

There are no conflicts to declare.

## Supplementary Material

RA-009-C9RA03235E-s001
