# Supplementary material for: First-principles computational investigation of nitrogen-doped carbon nanotubes as anode materials for lithium-ion and potassium-ion batteries
Source: RSC Adv. 2019 Jun 3;9(30):17299–307. doi: 10.1039/c9ra03235e (PMC9064600; doi:10.1039/c9ra03235e)
Supplement: RA-009-C9RA03235E-s001 [file RA-009-C9RA03235E-s001.pdf]

Electronic Supplementary Material (ESI) for RSC Advances.
This journal is © The Royal Society of Chemistry 2019

**Supporting information**

# First-principles computational investigation of nitrogen-doped carbon nanotubes as anode materials for lithium-ion and potassium-ion batteries

Chengxi Zhao[1,2], Yunxiang Lu[1]*, Honglai Liu[1], Linjiang Chen[2,3]*

[1]*Key Laboratory for Advanced Materials and Department of Chemistry, School of Chemistry and Molecular Engineering, East China University of Science and Technology, Shanghai, China*

[2]*Department of Chemistry and Materials Innovation Factory, University of Liverpool, Liverpool, UK*

[3]*Leverhulme Research Centre for Functional Materials Design, Materials Innovation Factory and Department of Chemistry, University of Liverpool, Liverpool, UK*

**Email: yxlu@ecust.edu.cn; linjiang.chen@liverpool.ac.uk*

(A)

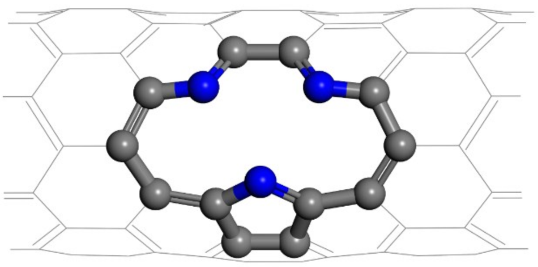

(B)

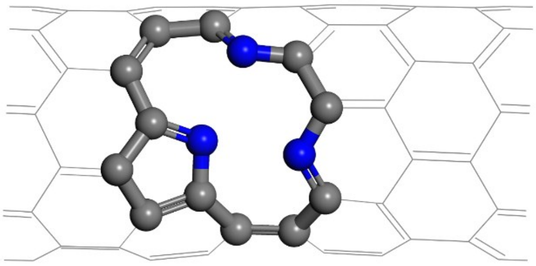

**Figure S1.** Two different types of N5. The formation energy of A is 0.7 eV lower than that of B.

**Table S1.** Charge transfer of one Li / K atom adsorbed on the N-doped CNTs (10,0).

| | Li | | K | |
|---|---|---|---|---|
| | outer surface | | inner surface | |
| | ΔQ (\|*e*\|) | ΔQ (\|*e*\|) | ΔQ (\|*e*\|) | ΔQ (\|*e*\|) |
| pristine | 0.88 | 0.89 | 0.90 | 0.96 |
| NQ | 0.87 | 0.90 | 0.90 | 0.96 |
| N6 | 0.84 | 0.84 | 0.87 | 0.89 |
| N5 | 0.85 | 0.82 | 0.87 | 0.89 |

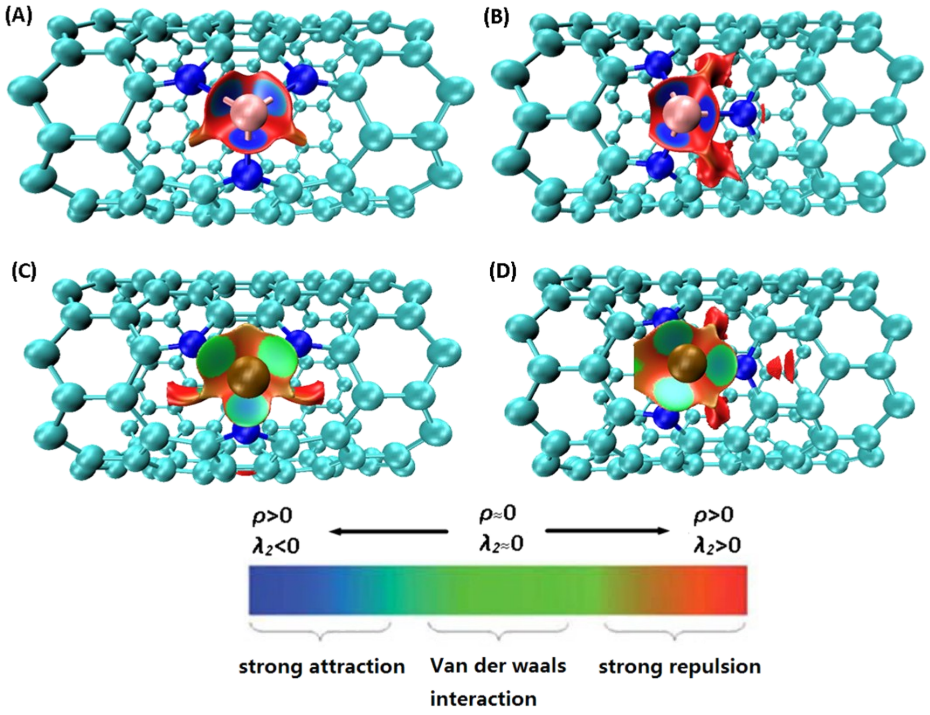


**Figure S2.** Non-covalent interaction index isosurfaces for (A) Li atom on N5 CNT (10,0), (B) Li atom on N6 CNT (10,0), (C) K atom on N5 CNT (10,0), (D) K atom on N6 CNT (10,0).

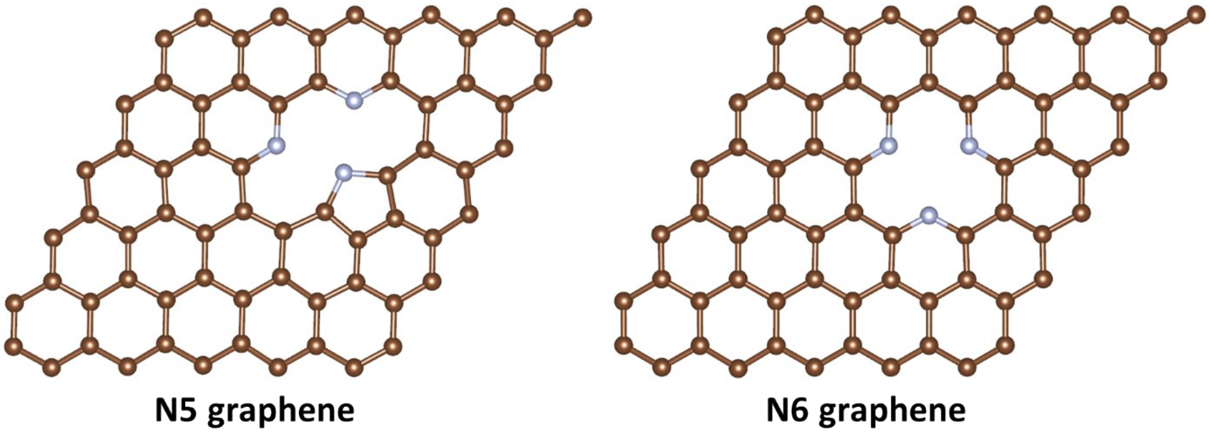


**Figure S3.** N5-doped and N6-doped graphene models.

Electronic density of states (DOS) of selected AM–NCNT systems is shown in Figure S3, with calculated Fermi levels set to zero (dash lines) and arrows representing the spin-up and spin-down states in the total DOS plots. The occupation numbers for spin-up and spin-down states are equal in the range of -20 to 5 eV, since all of the DOS plots have reflection symmetry about the line of zero state. We also calculated the partial density of states (PDOS). The overlap between the p orbitals of Li and the p orbitals of N is the direct evidence for the hybridization of these atoms. There also exists an overlap between the p orbitals of K and the p orbitals of N, albeit weaker than that between Li and N. Note that the p orbitals of K are mainly located at around -14 eV and are out of the scale in Figure S3 (F) and (H).

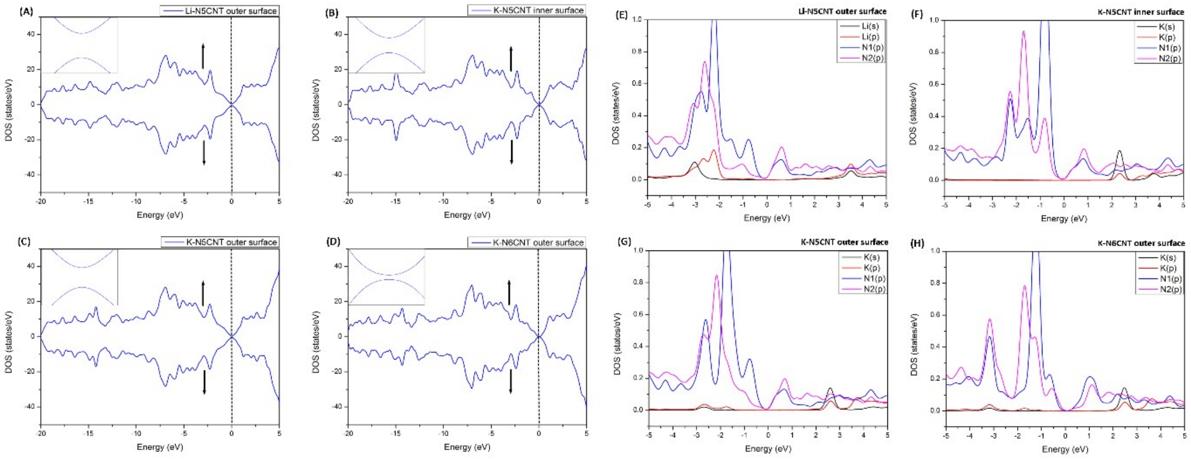


**Figure S4.** Total density of states (DOS) of (A) Li on N5 CNT outer surface, (B) K on N5 CNT inner surface, (C) K on N5 CNT outer surface, (D) K on N6 CNT outer surface; the inset in A–D shows the DOS curves near the Fermi level. Corresponding partial densities of states are shown in E–H. The Fermi energy level is set to zero (dashed lines).

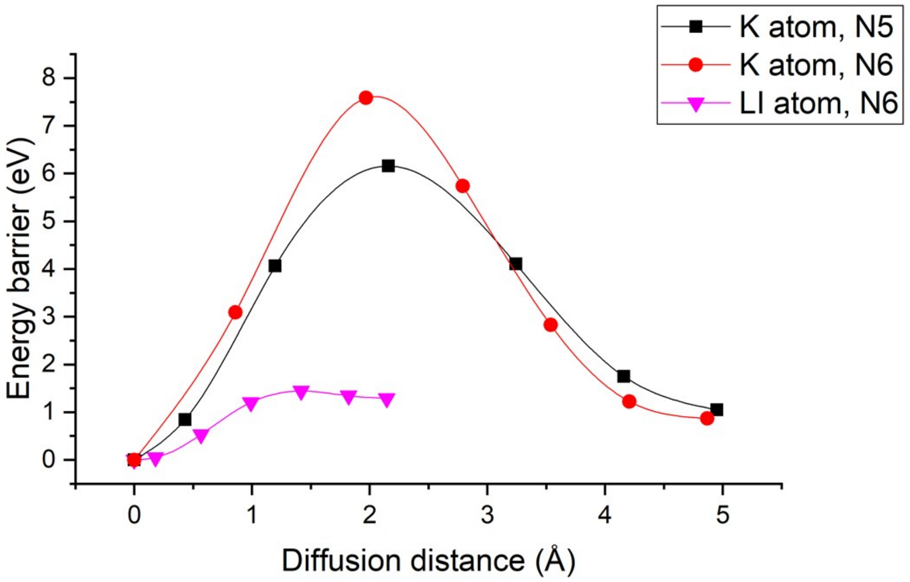


**Figure S5.** Potential energy curves for Li / K atom penetrating the sidewall of N5-doped and N6-doped CNTs (8,0).

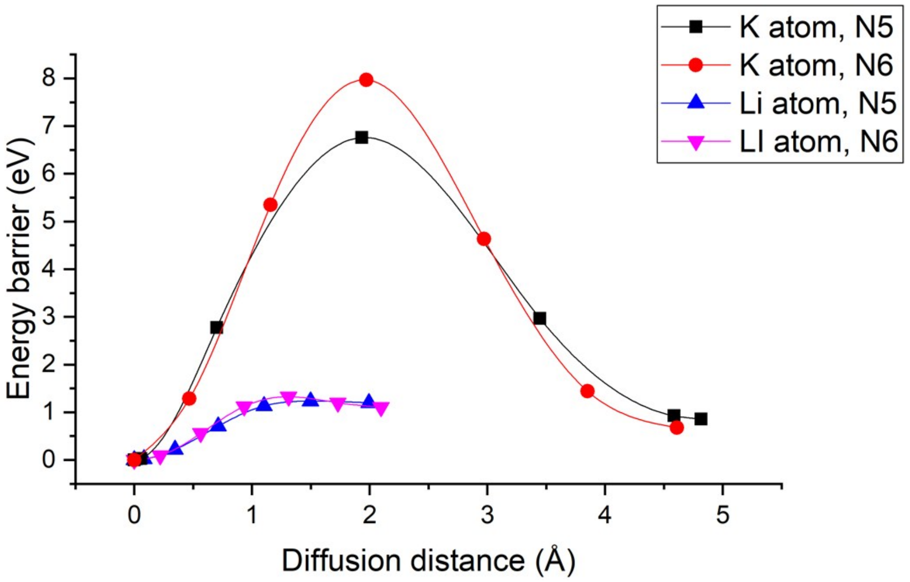


**Figure S6.** Potential energy curves for Li and K atom penetrate the sidewall of N5 and N6 doped CNT (12,0).

**References**


(1). Lu, T.; Chen, F. Multiwfn: a multifunctional wavefunction analyzer. J. Comput. Chem. **2012**, 33, 580−592.

(2). Humphrey, W.; Dalke, A.; Schulten, K. VMD: visual molecular dynamics. J. Mol. Graphics **1996**, 14, 33−38.
